# Recurrent primary cutaneous granular cell tumor of the neck in an Asian women: A case report

**DOI:** 10.1016/j.ijscr.2023.108213

**Published:** 2023-04-16

**Authors:** Ciniraj Raveendran, Ruby Elias, Sabu Parameswaran, I.P. Yadev

**Affiliations:** a Department of Radiation Oncology, Medical College Thiruvananthapuram, Kerala State, 695011, India; b Department of Pathology, Medical College Thiruvananthapuram, Kerala State, 695011, India; c Department of Plastic Surgery, Medical College Thiruvananthapuram, Kerala State, 695011, India; d Department of General Surgery, Medical College Thiruvananthapuram, Kerala State, 695011, India

**Keywords:** Granular cell tumor, Neck, Case report, Excision, Recurrence

## Abstract

**Introduction and importance:**

Granular cell tumors are uncommon neoplasms. They appear in the skin, subcutaneous tissues, and many internal organs. It is essential to diagnose this disease because it mimics other tumors clinically. We report this case because of the rarity of tumors in Asians and the necessity of excision with clear margins.

**Case presentation:**

A 55-year-old Indian woman reported swelling in the right side of the neck for six months and gradually increasing in size. Two years ago, she had a similar swelling excised from her neck. She had wide local excision of the tumor with wider margins in the plastic surgery department, and histopathology revealed a granular cell tumor with clear margins. She is undergoing close follow-up with history and physical examination with no evidence of disease recurrence.

**Clinical discussion:**

Granular cell tumors can have recurrences. These slow-growing tumors appear benign. Histopathological examination with careful assessment of high-risk features is vital in evaluating Granular cell tumors. Wide local excision with clear margins is the mainstay of treatment.

**Conclusion:**

Achieving clear margins in the head and neck area is sometimes tricky for granular cell tumors. Margin positivity is associated with a high risk of local recurrence and needs re-excision of the tumor for adequate local control.

## Highlights

• Granular cell tumors are uncommon in Asians.
• In the head or neck area, it may mimic the symptoms of other malignancies.
• Histopathology reveals polygonal, granular eosinophilic cells with vesicular nuclei and clumped chromatin.
• A positive margin means that local recurrence is likely to occur, and clear surgical margins are required to avoid this.

## 1 Introduction

Granular cell tumors (GCTs) are rare neoplasms, initially described by Russian pathologist Abrikossof as myoblastoma because of their similarity with granular cells of the skeletal muscle [1]. This tumor can occur in any body site, and recent studies with immunohistochemical markers show that they arise in the neuronal Schwann cells [2]. Most granular cell tumors are benign, some locally aggressive, with malignant changes seen in 0.5–2 % of the cases [3]. It is essential to recognize this disease histopathologically because these lesions often mimic common skin disorders [4]. The most common disease site is the skin and subcutaneous tissues, followed by the tongue, breast, esophagus, and vulva [5]. Benign lesions are usually <3 cm in size and appear insidiously, some of which regress spontaneously, too [6]. The best treatment for this tumor is local excision, irrespective of its benign or malignant nature [7]. It is unknown whether adjuvant therapy would benefit these tumors since radiation and chemotherapy are typically ineffective. Still, radiation therapy could help significantly if radical surgical resection restricts functional outcomes [8]. We report a granular cell tumor of the skin that was excised and recurred within two years of surgery. We discuss this case to highlight the importance of complete resection, the histopathological features, diagnosis, and therapeutic aspects of this rare tumor. We followed the SCARE guidelines when writing this case [9].

## 2 Case presentation

We report a case of a 55-year-old Indian woman, a housewife who presented in a tertiary care academic hospital in November 2021 with swelling on the right side of the neck for a 2-year duration. When she had similar swelling two years ago, she underwent a wide local excision in the general surgery department. Histopathology first revealed a granular cell tumor extending to the excision margin. She was lost to follow-up since the initial wide local excision two years back due to the financial difficulties in traveling for scheduled follow-ups. She has moderately controlled Type 2 diabetes mellitus using metformin and glimepiride. She has a history of thyroidectomy for colloid goiter years back. Her medical history shows she is normotensive and free of cardiovascular disease. She does not smoke or drink alcohol. There is no family history of this or any other type of cancer. She consulted the plastic surgery department to address the swelling on the right side of her neck.

**Fig. 1:**
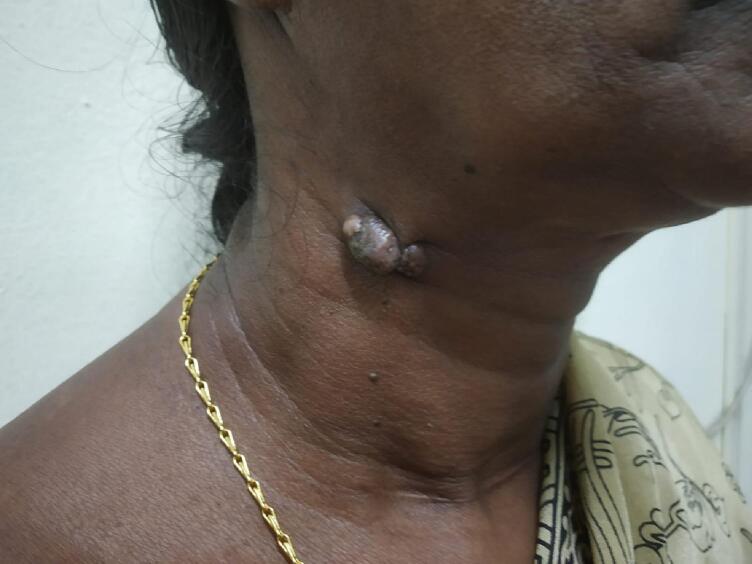
Photograph of the patient showing nodular swelling 3 × 3 cm on the right side of the neck.

The bulge gradually grew to 3 x3cm in length, nodular, mobile, and firm consistency (Fig. 1). There was no movement of swelling on protrusion of the tongue and deglutination. There were no similar swellings anywhere in the body. All the systemic examinations were within normal. Routine hematological investigations showed Hemoglobin, 12.1 g/dL, Total white blood cell count: of 8000 per mm^3^, Polymorphs: of 70 %, and Lymphocytes: of 30 %. Biochemical tests for blood sugars were Fasting Blood Sugar of 166 mg/dL and Post-Prandial Blood Sugar of 179 mg/dL. History, clinical findings, and a previous histopathology report of granular cell tumors reaching up to the resection margin raised high clinical suspicion of the recurrence of the granular cell tumor. In the plastic surgery department, she underwent wide local excision of the tumor on the right side of the neck in November 2021.

**Fig. 2:**
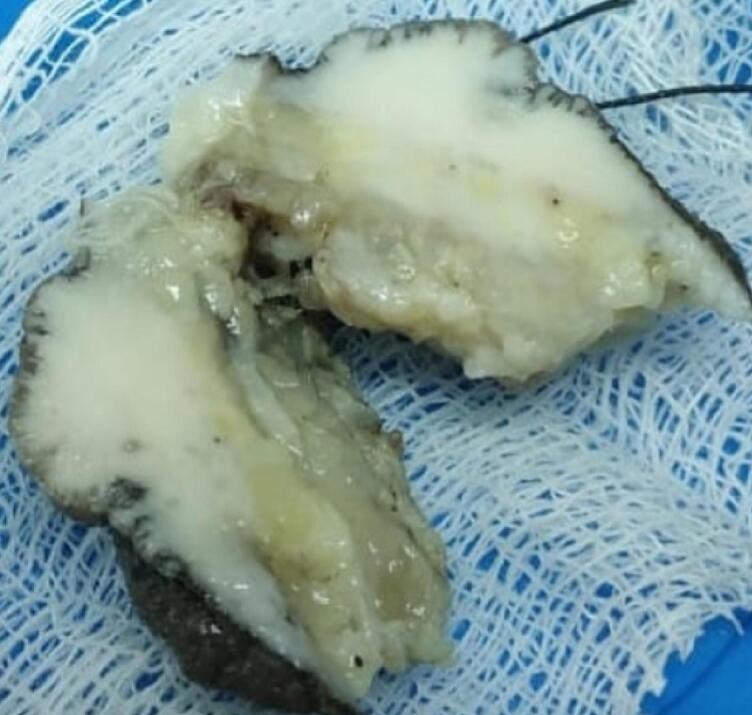
Macroscopy: Skin with an ill circumscribed grey white firm lesion (2.4 × 1.3 × 0.5 cm) in the dermis extending to the subcutaneous fat.

On gross examination, the skin and attached nodular tissue measured 4.5x2x1.3 cm. Surface skin measured 2.4 × 1.3 cm (Fig. 2). Surface skin showed a nodular lesion measuring 2.4 × 1.3 × 0.5 cm, situated 0.4 cm from the superior margin, 0.3 cm from the inferior margin, 0.7 cm from the lateral margin, and 1.2 cm from the medial margin. In addition, the cross-section identified a yellowish homogenous lesion corresponding to the surface nodularity situated 0.7 cm from the base with similar serial sections. In addition, there was skin with fibrofatty tissue that measured 2 × 0.8 × 0.2 cm cross-section of grey-white with no definite lesions.

**Fig. 3:**
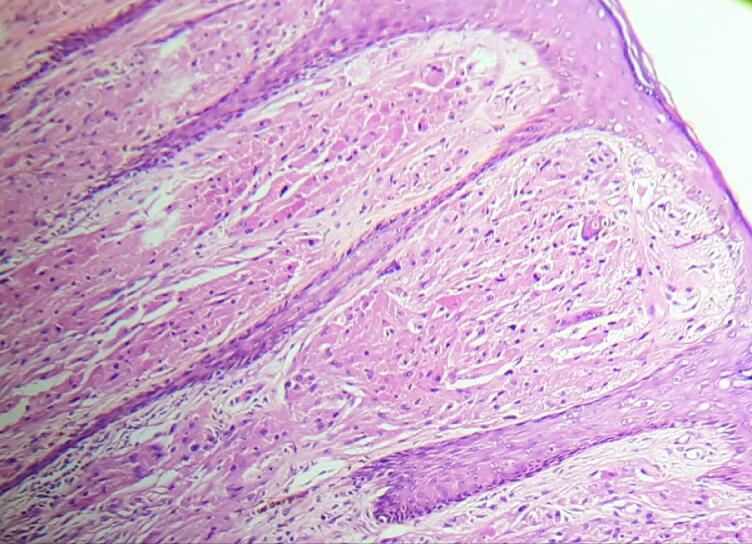
Pseudoepitheliomatous hyperplasia of the overlying skin Hematoxylin and Eosin, magnification 10×.

**Fig. 4:**
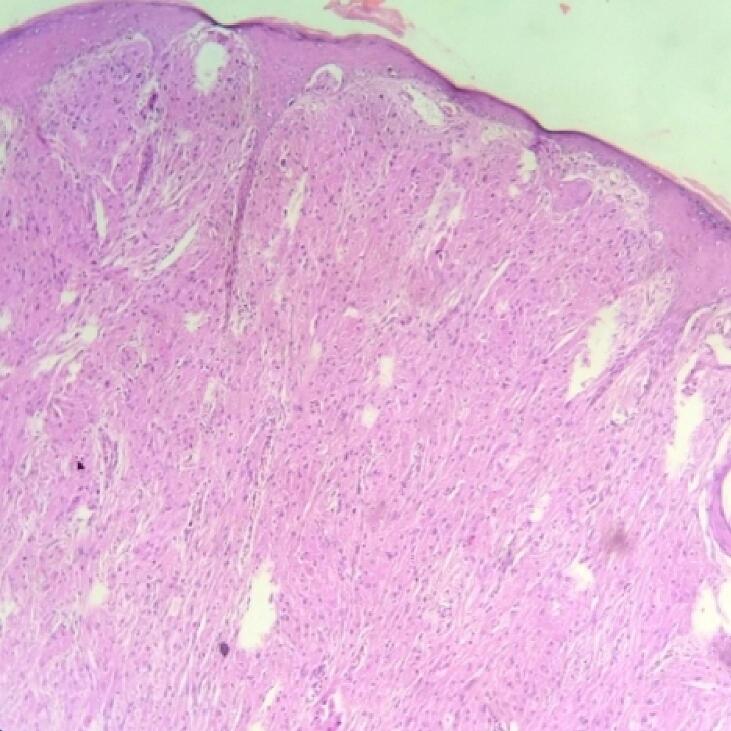
Skin with dermis and subcutis showing sheets of large cells Hematoxylin and Eosin, magnification 4×.

**Fig. 5:**
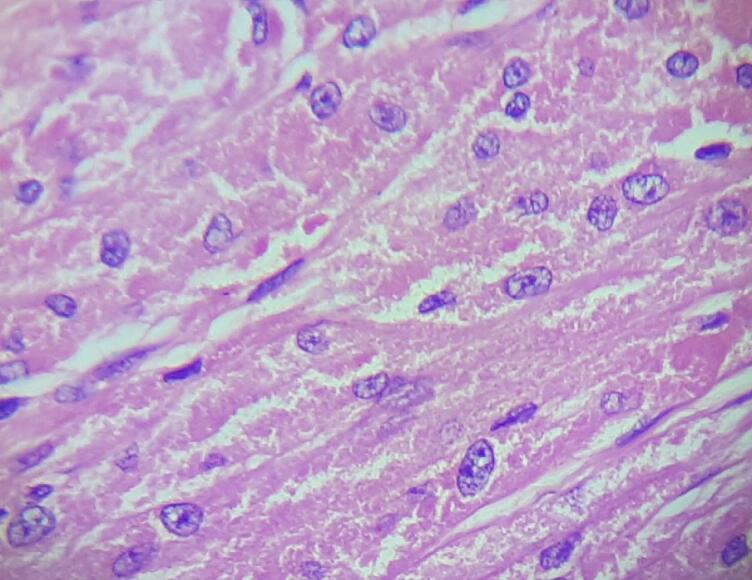
Large polygonal cells with abundant eosinophilic granular cytoplasm and round uniform nucleus Hematoxylin and Eosin, magnification 40×.

On microscopic examination, the section from the skin had papillomatous hyperplasia of the epidermis, with the dermis showing an infiltrating neoplasm composed of cells arranged in diffuse sheets and nests (Fig. 3, Fig. 4). The Individual cells were polygonal in shape with indistinct cell borders. The cells had abundant eosinophilic granular cytoplasm. There were round to oval vesicular nuclei with clumped chromatin and a few with conspicuous nucleoli (Fig. 5). There was no abnormal mitosis or necrosis, and the tumor cells were infiltrating subcutis. All these histopathological features are consistent with granular cell tumors. In addition, all margins, including the resection base, are neoplasm-free with a clearance of 0.7 cm. Unfortunately, we could not perform immunohistochemical analyses because of insufficient resources.

**Fig. 6:**
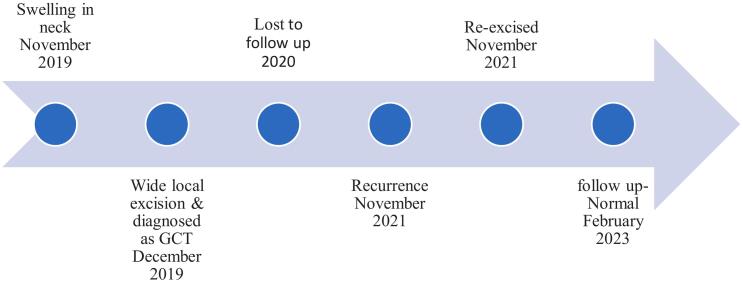
Timeline of events, interventions, and outcome (GCT- Granular Cell Tumor).

After this, she consulted the oncology department for further advice. Because of margin negativity and the benign nature of the disease, after an interdisciplinary discussion, we decided to keep her on close follow-up. As a result, she is being followed up in the oncology department every three months with a history and physical examination, with no evidence of disease recurrence during this short-term interval. In addition, the last follow-up visit on February 2023 for a general history and physical inspection of the local area was without any evidence of disease (Fig. 6).

## 3 Discussion

Granular cell tumors are uncommon neoplasms of neural origin, comprise <0.5 % of all soft tissue tumors, and often present as an asymptomatic nodule [10]. It can occur at any age, but the median age of presentation is 54 years, commonly affecting women [11], [12]. This tumor can occur in almost any body site, including the internal organs [13]. GCTs naturally occur close to areas where nerves are present. The affected areas are affected by the skin and subcutaneous tissues, oral mucosa, breast, ovaries, testis, and gastrointestinal tract [14]. These tumors are usually benign; malignancy is observed in <2 % of instances, most frequently affecting the extremities and the frequent site of metastasis being lymph nodes and lungs [15], [16]. Due to the depth of the lesion in the dermis, it can be challenging to make a preoperative diagnosis [17].

Our patient, a middle-aged woman in her fifties, presented with a slow-growing nodular skin lesion on the side of the neck. Because of the previous history of GCT in the same site, we did not make a preoperative attempt to diagnose by fine-needle aspiration cytology or trucut biopsy. Keeping a high clinical suspicion and possibility of recurrence, the surgical team planned for wide local excision with adequate margins. The definitive treatment for these cases is radical surgery with clear margins. In our case, the previous excision margins had been positive, which caused the tumor to recur. Surgical removal requires a negative margin to prevent local recurrence; however, it is unclear how much clearance is needed. There are a few reports where cases have been kept on follow-up after surgery, with positive margins, and the chance of local recurrence increases. Still, in benign GCTs, the rate of recurrences is low, even with margin positivity [11], [18]. Certain factors are associated with a high risk of malignancy associated with poorer survival, including the size of the lesion >5 cm and disease at distant sites [12]. Immediate recurrence of GCTs following surgery should raise the possibility of malignant transformation [19]. Our patient had a lesion size of <2.5 cm and a longer interval of two years between the first surgery and the current presentation; the chance of malignancy could be meager.

Fanburg Smith and colleagues used six histological criteria to clarify the malignant status and prognosis in patients with GCTs of soft tissues [3]. Their diagnostic criteria included tissue necrosis, spindling, vesicular nuclei with prominent nucleoli, high mitotic activity (>2 mitoses/10 high-power fields at 200× magnification), increased nuclear to cytoplasmic (N: C) ratio, and pleomorphism. In addition, they classified tumors based on the number of criteria met. Our patient was categorized as benign based on these histopathological criteria and not matching with any of these criteria.

Most benign GCTs are diagnosed based solely on histopathological examinations. The differential diagnosis of GCTs in the skin and subcutaneous include melanoma, lipoma, dermatofibrosarcoma, malignant fibrous histiocytoma, and sometimes chronic inflammation [20], [21], [22]. Frequently, histopathological analysis reveals large fascicles of tumor cells grouped in sheets or nests with pseudoepitheliomatous hyperplasia, infiltrating the collagen bundles into the dermis [23]. These tumor cells are large and polygonal and with variable cellularity; found singly, scattered, or in clusters, with indistinct cell borders and granular cytoplasm with an eccentric nucleus, small, binucleation, inconspicuous nucleolus with intranuclear inclusions in a background of grainy and stripped nuclei [22]. Taking into account our patient's histopathological characteristics, we made the diagnosis of benign GCT. Immunohistochemical stains frequently used to diagnose these tumors often stain positive for S-100, CD 56, CD 58, inhibin – α, neuron-specific enolase, and Ki-67, with a preference for specific markers depending on the location of the tumor [24], [25]. S-100 and pan-cytokeratin are also commonly used, with tumor cells staining positive for S-100 but negative for cytokeratin. We have not used immunohistochemical markers because of the previous diagnosis of GCT in this case, diagnostic criteria fitting into a benign GCT, and limited resources for performing these tests.

The treatment of GCT is surgery; wide local excision of the tumor with negative margins is the top priority. Unfortunately, our knowledge of the margin clearance required for this tumor is limited. Therefore, GCTs are not highly aggressive, even though a local tumor extension is common, and such highly complex surgical procedures are not indicated [26]. Sometimes conservative treatment is advised if the tumor is close to vital structures, and removal of which is associated with excessive mortality or morbidity [27]. Historically, these tumors have been resistant to radiotherapy. Nevertheless, radiation treatment is essential in inoperable or incomplete surgical excision, especially in proton beam treatment where revision surgery is impractical due to technical constraints or anticipated functional morbidity.

Nevertheless, radiation treatment is essential in inoperable or incomplete surgical excision, especially in proton beam treatment where revision surgery is impractical due to technical constraints or anticipated functional morbidity [28]. The role of chemotherapy is limited to metastatic GCTs, often associated with poor survival [29]. Pazopanib monotherapy is associated with some objective responses in patients [30].

## 4 Conclusion

Granular cell tumors are rare neoplasms of the skin and subcutaneous area. It should be a differential diagnosis of slow-growing tumors in the head and neck area. Careful histopathological examination and looking for high-risk features are essential for diagnosis. Surgery with wide local excision and clear margins is the mainstay of treatment. Microscopic disease at the margins is associated with recurrence. *Re*-excisions can be technically challenging in difficult areas like the head and neck. Re-excision with wider margins is a therapeutic option in recurrent GCTs.

## Patient perspective

I had a bump on my neck two years ago. I saw it accidentally and was a little worried as it grew bigger. I talked to my doctor, and he told me to get it removed, so I went to the hospital for surgery. Afterward, I didn't care about it. I had it taken away. I was more worried when the swelling started to grow again in the same place and the same way it had before. I saw the specialist doctor, who said I should have another surgery. I've had it taken out, and now I'm happy.

## Informed consent

Written informed consent was obtained from the patient to publish this case report and accompanying images. A copy of the written consent is available for review by the Editor-in-Chief of this journal at request.

## Data and materials

Not applicable.

## Provenance and peer review

Not commissioned, externally peer-reviewed.

## Ethical approval

Ethics approval is not required for publishing case reports in our institution.

## Funding

There is no funding for this research.

## Author contribution

Ciniraj Raveendran conceived the idea, did the literature search, and prepared the initial draft. Ruby Elias was involved in the diagnosis, designed the photomicrograph, and contributed to manuscript preparation. Sabu Parameswaran was involved in data collection, the treatment and care of the patient, and assisted in draft preparation. Finally, IP Yadev was involved in revising the manuscript and editing the final version. All authors read and approved the final manuscript.

## Guarantor

Dr Ciniraj Raveendran

## Conflict of interest statement

All authors declare no conflict of interest.
